# A Biomimetic Chitosan Derivates: Preparation, Characterization and Transdermal Enhancement Studies of *N-*Arginine Chitosan 

**DOI:** 10.3390/molecules16086778

**Published:** 2011-08-09

**Authors:** Hui-Xia Lv, Zhen-Hai Zhang, Xiao-Pan Wang, Qing-Qing Cheng, Wei Wang, Xu-Hui Huang, Jian-Ping Zhou, Qiang Zhang, Lu-Lu Hou, Wei Huo

**Affiliations:** 1 Department of Pharmaceutics, China Pharmaceutical University, No. 24 tongjiaxiang Nanjing, China; Email: lvhuixia@163.com (H.-X.L.); 2 Jiangsu Province Academy of Traditional Chinese Medicine, Nanjing, No. 100 shizhijie Nanjing, China; Email: davidpharm@yeah.net; 3 Department of Pharmacy, Fujian Provincial Hospital, China. No.134 dongjie Fuzhou, China; 4 State Key Laboratory of Natural and Biomimetic Drugs, School of Pharmaceutical Sciences, Peking University, No.38 Xueyuan Road Haidian District, Beijing, China; Email: zhangqiangdodo@bjmu.edu.cn; 5 Jiangxi Xierkangtai Pharmaceutical Co. Ltd., North Zone, High-New Technology Industrial Zone, Pingxiang, Jiangxi, China; Email: hoululu_12345@hotmail.com (L.-L.H.)

**Keywords:** biomimetic chitosan derivates, arginine-rich, cell penetration peptides, *N-*arginine chitosan, transdermal enhancer, adefovir

## Abstract

A novel arginine-rich chitosan (CS) derivates mimicked cell penetration peptides; *N-*Arginine chitosan (*N-*Arg-CS) was prepared by two reaction methods involving activated L-arginine and the amine group on the chitosan. FTIR spectra showed that arginine was chemically coupled with CS. Elemental analysis estimated that the degrees of substitution (DS) of arginine in CS were 6%, 31.3% and 61.5%, respectively. The drug adefovir was chosen as model and its permeation flux across excised mice skin was investigated using a Franz diffusion cell. The results showed that the most effective enhancer was 2% (w/v) concentration of 10 kDa *N-*Arg-CS with 6% DS. At neutral pH, the cumulative amount of adefovir permeated after 12 hours was 2.63 ± 0.19 mg cm^−2^ which was 5.83-fold more than adefovir aqueous solution. Meanwhile *N-*Arg-CS was 1.83, 2.22, and 2.45 times more effective than Azone, eucalyptus and peppermint, respectively. The obtained results suggest that *N-*Arg-CS could be a promising transdermal enhancer.

## 1. Introduction

Cell-penetrating peptides (CPPs) are short cationic peptides such as Transactivator of Transcription (TAT), penetratin and oligoarginine that facilitate the cellular uptake of various molecular cargos (from small chemical molecules to nanoparticles and large fragments of genes or proteins). CPPs typically have an amino acid-based composition that contains positively charged amino acids such as arginine or lysine. In the case of oligoarginine and related peptides, the salient structural feature is that they are very rich in arginin in general. There has been no real consensus as to the mechanism of CPP translocation, but there was no doubt that an arginine with a guanidinium group is critical to the translocation process [1,2]. Since arginine is abundant in cell-penetrating peptides which are highly cationic, they strongly adsorb on the membrane surfaces via hydrogen bond-induced formations of the guanidino moieties in arginine with anionic phosphates, sulfates, and carboxylates of cellular components [3]. 

Notably, chitosan (CS) is a nontoxic biopolymer that are produced by the deacetylation of chitin, and currently CS and its derivatives are receiving considerable attention in pharmaceutical and commercial applications due to their biological activities and properties [4,5,6,7]. Because of their permeation enhancing effect, enzyme inhibitory capabilities, mucoadhesive properties [8], CS and its derivatives are important excipients for delivery systems. 

In this study, *N-*Arg-CSs, which we call biomimetic chitosan derivatives, prepared by chemical coupling of L-arginine, mimicked the argnine-rich peptides in order to get the cellular uptake functions of typical CPPs. *N-*ArgCSs were first reported in 2004 as novel anticoagulant biomaterials [9]. From then on some other functions of *N-*Arg-CS were investigated. These functions include antibacterial action [10,11], gene transfection efficiency enhancement [12] and siRNA delivery [13]. Meanwhile our previous study (not published yet) has shown that *N-*Arg-CS can effectively enhance the oral absorption and the transdermal delivery of some drugs. To date, no report has ever describved the above function of *N-*Arg-CS. 

Adefovir [9-(2-phosphonomethoxyethyl) adenine] is an acyclic nucleoside phosphonate used as a broad-spectrum antiviral that is highly effective against herpes-, retro-, and hepadnaviruses. Meanwhile other studies have shown that a 1–5 mg dosage of adefovir is effective against filterable virus which causes skin disease [14]. The major drawbacks of adefovir with regard to oral administration are its low oral bioavailability, dose-dependent nephrotoxicity and gastrointestinal disturbance. Transdermal drug delivery offers numerous advantages over conventional routes of administration, by supplying a sustained release of drug to provide a steady plasma profile and hence reduce side effects, avoid the first-pass metabolism and circumvent the gastrointestinal tract. The aforementioned facts show that transdermal delivery of adefovir might be necessary as an alternative to the oral administration. Inspired by the aforementioned research work we have prepared a series of *N-*Arg-CS with different molecular weights and DS values and explored their transdermal enhancement of adefovir absorption.

## 2. Results and Discussion

### 2.1. Characterization of N-ArgCS samples

**Figure 1:**
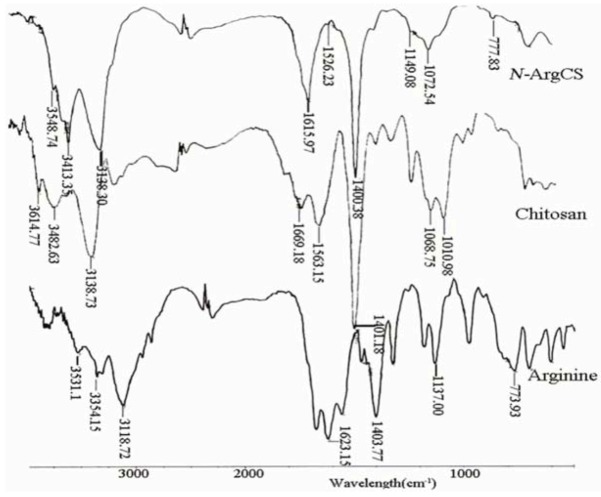
FTIR spectrums of the CS, L-Arg and *N-*Arg-CS-B.

The FTIR spectra of L-Arg, CS and *N-*Arg-CS-B samples are shown in Figure 1. For arginine, the absorption band at 1623.15 cm^−1^ is assigned to the guanido group, and the band at 1403 cm^−1^ is attributed to COO^–^ symmetric bending. The C-C-N asymmetric bending and COO^–^ scissioning modes are found at 1137 cm^−1^ and 773 cm^−1^, respectively [15,16]. Chitosan exhibits the characteristic bands of NH_2_ scissoring vibrations at 1669 cm^−1^, carbonyl asymmetric stretching vibrations at 1563 cm^−1^, and C-O stretching vibrations of the pyranose ring at 1068 cm^−1^ and 1010 cm^−1^ [17]. Comparing with those of chitosan and arginine, several noticeable changes are apparent in the spectra of *N-*Arg-CS samples. The band of guanido group appears at 1615.97 cm^−1^ and the band of C-C-N asymmetric bending at 1149 cm^−1^. The new band at 1526.23 cm^–1^ is most likely due to an amide bond linking chitosan and arginine [9].

The substitution degrees of arginine in CS estimated from elemental analysis were about 6% (*N-*Arg-CS-A, *N-*Arg-CS-B, and *N-*Arg-CS-C), 31.3% (*N-*Arg-CS-D) and 61.5% (*N-*Arg-CS-E), respectively, as presented in Table 1. 

**Table 1:** The elemental analysis of CS and *N-*Arg-CS.

Samples	MW	C(%)	N(%)	C/N	DD(%)	DS(%)
CS	10kD	32.12 ± 0.02	6.06 ± 0.05	5.30	91.0	-
*N*-ArgCS-A	5kD	36.68 ± 0.01	8.17 ± 0.03	4.49	-	6.3%
*N*-ArgCS-B	10kD	36.52 ± 0.03	8.08 ± 0.05	4.52	-	6.0%
*N*-ArgCS-C	20kD	36.56 ± 0.06	8.07 ± 002	4.53	-	5.9%
*N*-ArgCS-D	10kD	37.64 ± 0.02	12.26 ± 0.08	3.07	-	31.3%
*N*-ArgCS-E	10kD	48.95 ± 0.04	19.98 ± 0.06	2.45	-	61.5%

*Notes*: MW is molecular weight; DD is the degree of deacetylation; DS is the degree of substitution.

**Figure 2:**
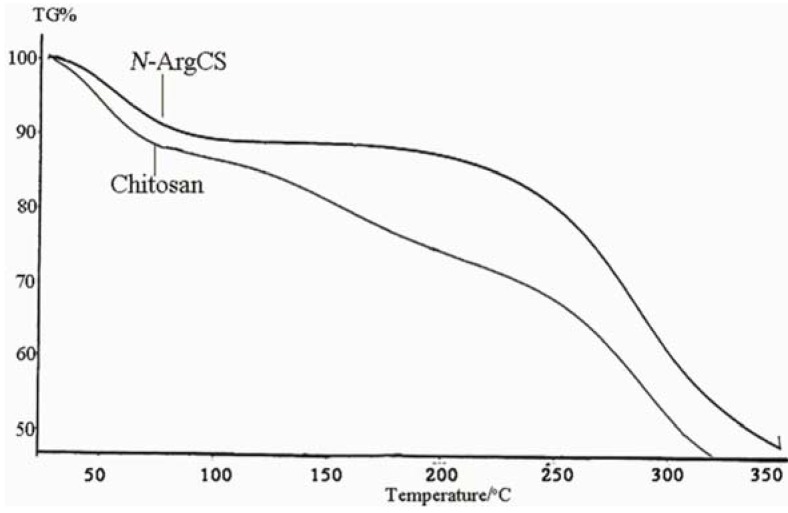
TG thermogravimetric of CS and *N-*Arg-CS-B.

TG curves of chitosan and *N-*Arg-CS samples are shown in Figure 2. The thermogram of *N-*Arg-CS has three weight loss stages. The first stage ranges between 30 and 90 °C and shows about 11.15% loss in weight, corresponding to the evaporation of adsorbed and bound water. The second one starts at 90 °C and continues up to 180 °C during which there is no significant weight loss. The weight is abruptly decreased when the temperature reaches 250 °C due to the degradation of *N-*Arg-CS together with the breakage of the amide linkage of *N-*Arg-CS. Compared to *N-*Arg-CS, the first stage of chitosan decomposition occurs between 0 and 60 °C, associated with the loss of bound water in the samples. The second one ranging from 60 to 300 °C corresponds to further dehydration and degradation of the samples. 

### 2.2. In vitro skin permeation

#### 2.2.1. Skin penetration of Adefovir with *N-*Arg-CS of different MWs in various pH values

Table 2 presents the accumulative percutaneous amount (Q) and steady-state permeation rate of adefovir with 60.0 mg *N-*Arg-CS of different MWs in 3 mL adefovir solutions (containing 6.0 mg of adefovir) at various pH values. 

**Table 2:** Effect of *N-*Arg-CS at various pH values on the skin penetration of adefovir.

Samples	pH	Qn(µg·cm^–2^)	Jss(µg·cm^−2^·h^−1^)
Control	3.0	457.08 ± 17.15	37.70
	5.0	457.69 ± 21.00	38.16
	7.0	450.82 ± 23.02	35.53
	9.0	330.65 ± 24.38	26.75
*N-*Arg-CS-A	3.0	1714.07 ± 7.79	144.23
	5.0	1826.66 ± 28.61	148.61
	7.0	2125.53 ± 118.48	173.53
	9.0	1815.55 ± 39.10	149.32
*N-*Arg-CS-B	3.0	2306.56 ± 23.61	189.47
	5.0	2333.18 ± 15.31	191.60
	7. 0	2628.86 ± 71. 50	208.87
	9. 0	2318.23 ± 26. 54	191.35
*N-*Arg-CS-C	3.0	1386.08 ± 33. 82	109.52
	5.0	1388.28 ± 141.75	110.69
	7.0	2064.91 ± 65. 13	169.17
	9.0	1861.52 ± 24. 11	149.78

The results showed that both transdermal permeation profile of adefovir with or without *N-*Arg-CS followed zero-order kinetics. Meanwhile, compared to those of adefovir solutions without enhancer, both the Q and J_ss_ of adefovir solutions with 2% *N-*Arg-CS-A, *N-*Arg-CS-B, *N-*Arg-CS-C as enhancer, respectively, had significant difference (P < 0.05). The effects of different *N-*Arg-CS specimens depended on the pH. As for *N-*Arg-CS-A and *N-*Arg-CS-B, the cumulative percutaneous amount increased in the order Q_pH3_ < Q_pH5_ ≈ Q_pH9_ < Q_pH7_, and Q_pH7_ is higher than other three Qs (P < 0.05). However, the result of *N-*Arg-CS-C is a little different with the order Q_pH3_ ≈ Q_pH5_ < Q_pH9_ < Q_pH7_ and Q_pH7_ is also higher than other three Qs (P < 0.05). This result supports the observation of Vávrova [18,19].

The results obtained were likely due to different dissociation states of adefovir and *N-*Arg-CS in at the different pH values. Adefovir is an ionic compound with three pK_a_s, 1.2 (loss of a proton from the dihydrogenphosphonate and formation of a zwitterion), 4.2 (release of the free base at N1 and formation of a monoanion) and 6.8 (formation of a phosphonate dianion) [20]. At pH 7 adefovir exists in the form of a monoanion and a dianion with an almost equal 1:1 molar ratio. *N-*Arg-CS is a positively charged polymer due to the existence of a different state of the guanido group of arginine in acidic, neutral and basic solutions. A complex of *N-*Arg-CS and adefovir might be formed by the interaction between positive and negative charge to enhance drug transdermal delivery. 

Meanwhile different molecular weight *N-*Arg-CS with the same DS had different accumulative percutaneous amounts. The results showed that the accumulative percutaneous amount with *N-*Arg-CS-B (Mw, 10 kDa) was higher than that of *N-*Arg-CS-A (Mw, 5kDa) and *N-*Arg-CS-C (Mw, 20 kDa) under all pH circumstances. 

#### 3.2.2. Effect of *N-*ArgCS DS and concentration on the skin penetration of adefovir

**Figure 3:**
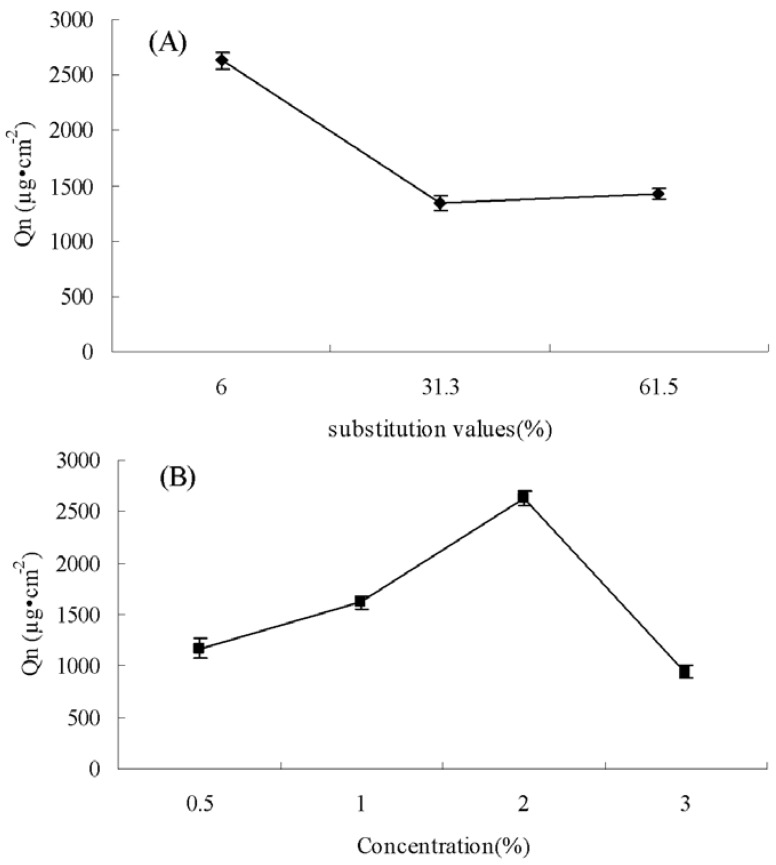
Effect of *N-*Arg-CS with different substitution values (**A**) and concentrations (**B**) on the skin penetration of adefovir after 12 h (n = 6) (A: The samples are *N-*Arg-CS-B, *N-*Arg-CS-D and *N-*Arg-CS-E and the concentration is 2%; B: The sample is *N-*Arg-CS-B).

The effect of *N-*Arg-CS substitution value on adefovir transdermal delivery is presented in Figure 3(A). The results showed that the cumulative percutaneous amount with *N-*Arg-CS-B was significantly higher than that of *N-*Arg-CS-D and *N-*Arg-CS-E (P < 0.05). Meanwhile, there is no significant difference between *N-*Arg-CS-D and *N-*Arg-CS-E (P > 0.05). It was unexpected that the increase in DS of the *N-*Arg-CSs did not enhance their penetration properties.

Regarding the use of different concentrations of *N-*Arg-CS, the situation is the same as in the case of DS. The increase in concentration of *N-*Arg-CS did not lead to an increase in the penetration enhancement properties of *N-*Arg-CS as presented in Figure 3(B). 2% of *N-*Arg-CS seemed to be the optimum concentration.

#### 2.2.3. Comparison of different enhancers

**Figure 4:**
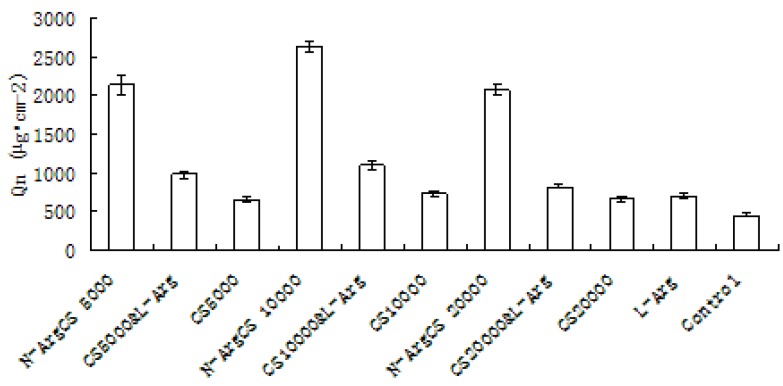
The accumulated transportation of adefovir across mice skins with various enhancers after 12 h (n = 6).

Table 3 and Figure 4 present the accumulative percutaneous amount (Q) of adefovir with 60 mg arginine, different MW chitosans, physical mixtures of arginine and chitosan (1:1) and *N-*Arg-CS in 3 mL natural pH adefovir solution (contains 6.0 mg of adefovir).

**Table 3:** The accumulated transportation of adefovir across mice skins with various enhancers after 12 h (n = 6).

Enhancer	Qn/µg·cm^−2^	Jss/µg·cm^−2^·h^−1^
Control	450.82 ± 23.02	35.53
L-Arg	701.16 ± 27.99	60.13
CS5000	652.41 ± 29.65	51.83
CS10000	729.82 ± 35.45	55.08
CS20000	660.89 ± 21.02	59.13
Mixture of CS5000& L-Arg	975.12 ± 47.37	79.13
Mixture of CS10000& L-Arg	1097.58 ± 49.88	93.30
Mixture of CS20000& L-Arg	819.91 ± 26.08	64.02
*N-*Arg-CS-A	2125.53 ± 118.48	173.53
*N-*Arg-CS-B	2628.86 ± 71.50	208.87
*N-*Arg-CS-C	2064.91 ± 65.13	169.17
Azone	1439.04 ± 59.51	124.59
Eucalyptus	1182.55 ± 76.43	98.47
Peppermint	1072.81 ± 55.82	73.51

The results show that the cumulative amount of adefovir with arginine, different MWs CS and the physical mixture of arginine and CS after 12 hour were 1.53, 1.43, 1.60, 1.44, 2.13, 2.40, 1.79 folds more than control sample, respectively. The transdermal enhancement of the arginine is likely due to its guanidino group. It was reported that guanidino group had the ability to open the tight junctions between cells and to reduce the membrane potential [21]; the chitosan can open the tight junctions of the epithelia [8]. The enhancement of arginine and CS mixture could be of the addition effect of both materials. Furthermore the cumulative amount of adefovir with *N-*Arg-CS-A, B, C after 12 hours was 4.71, 5.83, 4.58 times that of the control, respectively The above shows that *N-*Arg-CS, chemical coupling of arginine and CS could be an promising transdermal enhancer for adefovir. 

**Figure 5:**
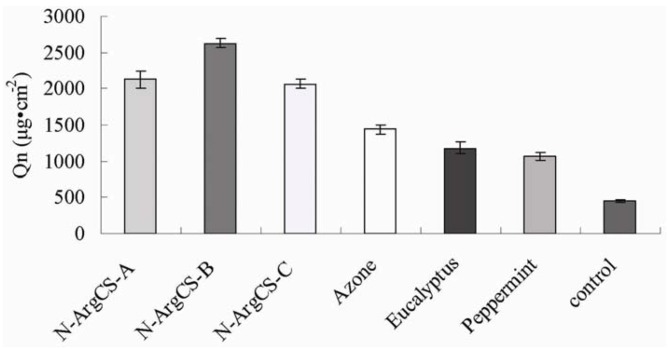
The accumulated transportation of adefovir across mice skins with various enhancers after 12 h (n = 6).

Azone (1-dodecylazacyclohepta*N-*2-one or laurocapram) was the first molecular that was specifically designed as a skin penetration enhancer. It probably exerts its penetration enhancing effects through the interactions with the lipid domains of the *stratum corneum*. Eucalyptus and peppermint are essential oils, and they can impact membrane fluidity to exert their penetration enhancement [22]. Comparison of N-Arg-CS enhancement with that provided by azone, eucalyptus and peppermint is presented in Table 3 and Figure 5. The results show that cumulative amount drug permeated by azone, eucalyptus and peppermint after 12 hours was 2.14, 1.58, 1.34-fold more than control, respectively. The enhancement of *N-*Arg-CS-B was found to be 1.83, 2.22, 2.45 folds more than that of azone, eucalyptus and peppermint, respectively. These results show that *N-*Arg-CS is slightly superior to typical penetration enhancers.

## 3. Experimental

### 3.1. Materials

CS (MW5 kDa, 10 kDa, 20 kD, DD > 90%) from shrimp was purchased from Golde*N-*shell Biochemical Co., Ltd. (Zhejiang, China). L-Arg was purchased from Shanghai HuiXing Biochemistry Reagent Limited Company. (Shanghai, China). Adefovir was purchased from Nanjing Chia-Tai Tianqing Pharmacy Co. Ltd. (Nanjing, China). All the other chemicals were of analytical grade and can be used without further purification. Healthy male Kunming species mice (25–30 g) were supplied by Qinglongshan Laboratory Animal Center. (Nanjing, China). All animal experiments complied with the rules that are set forth in the NHI Guide of the Care and Use of Laboratory Animals. 

### 3.2. Synthesis of N-ArgCS with different DS

#### 3.2.1. Synthesis of *N-*ArgCS with low DS

**Scheme 1:**
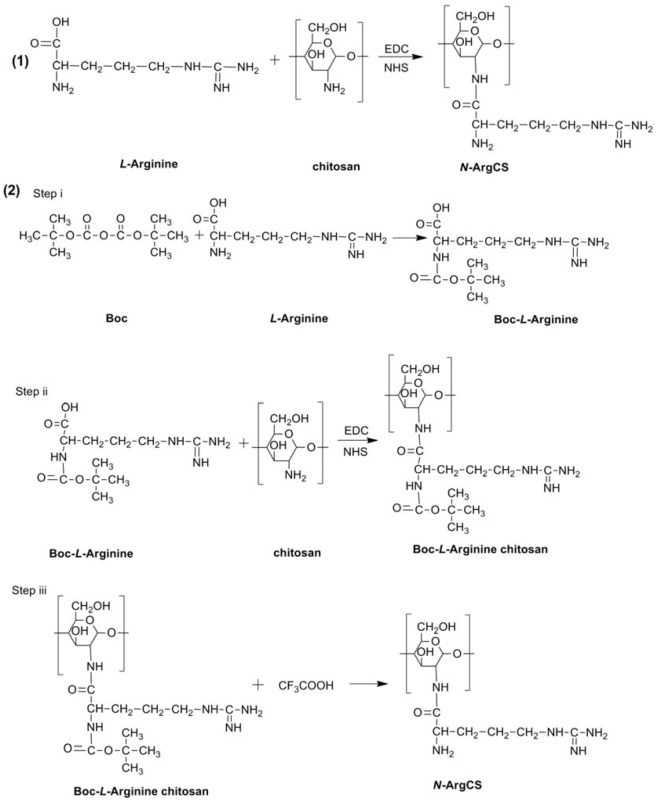
Two methods of *N-*ArgCS synthesis.

The *N-*ArgCS samples with low DS were synthesized by a modified method described elsewhere [9], as shown in the Scheme 1(1). First L-Arg (0.3 g) was dissolved in distilled water (10 mL). *N-*hydroxy-succinimide (NHS) and 1-ethyl-3-(3-dimethylaminopropyl) carbodiimide (EDC) were added to the solution, respectively, at a fixed molar ratio of 1:1:3, to activate the carboxyl group of L-Arg. The pH of the resultant solution was adjusted to 6 with 1% acetic acid and 1% NaOH solution. The carboxyl group of L-Arg was activated for 2 h. Secondly; chitosan (1 g, MW 5 kDa) was dissolved in 1% acetic acid solution (100 mL). The pH was adjusted in the same manner as L-Arg solution with 1% NaOH solution. The activated L-Arg solution was added to the chitosan solution to react at ambient temperature with continuous stirring for 48 h. The resultant solution was dialyzed (MWCO = 3,500) against distilled water for 48 h, then lyophilized. The final product was named as *N-*ArgCS-A. Using the same method for preparation of *N-*ArgCS-A, and other chitosans (MW 10 kDa and 20 kDa) *N-*ArgCS-B and *N-*ArgCS-C, were produced, respectively. 

#### 3.2.2. Synthesis of *N-*ArgCS with high DS

In order to increase the DS, dicarbonic acid bis (1,1-dimethylethyl)ester (Boc 20) was used to prevent the reaction of activated L-Arg molecules with each other, as shown in Scheme 1(2). L-Arg (0.44 g) was dissolved in distilled water (20 mL). Boc (0.54 g) was dissolved in tetrahydrofuran (THF, 20 mL), and then added to the L-Arg solution. The pH was adjusted to 6 with 1M NaOH solution and allowed to react at ambient temperature for 24 h. The product was isolated by removing the THF under reduced pressure using a rotary evaporator. The final product from the evaporation process was dissolved again in distilled water (20 mL). NHS and EDC were added at fixed molar ratio of 1:1:3 (g/g/g) as L-Arg/NHS/EDC, respectively. The pH of the resultant solution was adjusted to 6 with 1% acetic acid and 1% NaOH solution. The Boc carboxyl group of the protected L-Arg was activated for 2 h. Exactly 1 g of chitosan (MW 10 kDa) was dissolved in 1% acetic acid solution (100 mL) and the pH was adjusted to 6. The activated Boc protected L-Arg solution was added into chitosan solution to react at ambient temperature with continuous stirring for 48 h. After the reaction was complete, the Boc groups were deprotected with 1% CF_3_COOH. The obtained solution was dialyzed (MW cutoff = 3,500) against distilled water for 48 h and then lyophilized. The final product was named as *N-*Arg-CS-D. *N-*ArgCS-E was prepared in the same manner with the ratio of 1:1.31:1.63 (g/g/g, CS/L-Arg /Boc). 

### 3.3. Characterization of N-ArgCS

FTIR spectra of chitosan, L-Arg and *N-*ArgCS-B were measured by using a FT-IT Nicolet Impact 410 spectrophotometer. Powder samples were mixed with KBr, and pressed into disks for measurement. The DS of *N-*Arg-CS sample was determined by elemental analysis (C, N) using a Vario EL III elemental analysis instrument. Thermal degradation (TG) of samples was monitored using a thermal analyzer. Chitosan and *N-*ArgCS-B were heated from ambient temperature to 400 °C at a constant heating rate of 10 °/min under a nitrogen atmosphere. 

### 3.4. In vitro permeation experiments

Abdominal skins were obtained from male Kunming species mice weighing 25–30 g. After its hair was shaved carefully with an electric clipper, the skin was excised from the abdominal region of each scarified mouse and the subcutaneous fat and other extraneous tissues were trimmed out with physiologic saline. The excised mice skins were washed, then stored at 4 and used within 12 h after the skin was harvested. The permeation experiments were performed using Franz diffusion cells fitted with excised mice skins at 32 °C. The effective diffusion area was 1.54 cm^−2^ and the receptor chamber was filled with 16 mL of physiologic saline which was constantly stirred at 400 rpm throughout the experiment. After adefovir and *N-*Arg-CS mixture solution was applied on the epidermal surface of the skin, 2 mL of medium in receptor chamber was withdrawn at specific time intervals for up to 12 h. An equal volume of the fresh physiologic saline was immediately replenished after each sampling process. Collected samples were filtered through 0.22 μm microporous membrane filter and adefovir was quantified by HPLC analysis. 

### 3.5. Drug analysis

Samples were analyzed by LC - 10AT VP HPLC system with a SPD - 10A VP variable-wavelength ultraviolet absorbance detector and a reverse phase Licrosphere C_18_ column (250 mm × 4.6 mm i. d, 5 µm) operating at room temperature. The sample (1 mL) was precipitated by 1 mL methanol and filtered by using 0.22 μm filter membrane. The mobile phase composed of a mixture of methanol/phosphate buffer at pH 2.5 in a ratio of 80:20 v/v, respectively, the flow rate was 0.5 mL·min^−1^. Adefovir was detected at 261 nm with the retention time of 5.3 min. The standard curve was linear over the concentration range 1–90 µg·mL^−1^ (R^2^ = 0. 9999). The coefficients of variation (RSD) for inter- and intraday variation was both below 1%. 

### 3.6. Data analysis

The cumulative amount of adefovir permeating through the skin was plotted as a function of time. The skin flux was determined from Fick’s law of diffusion: J_ss_ = dQ_n_/Adt. Where, J_ss_ is the steady-state skin flux in μg·cm^−2^·h^−1^, dQ_n_ is change in quantity of the drug passing through the skin into the receptor chamber in dt hours, A is the active diffusion area in cm^2^ and dt is the change in time [23]. The cumulative amount of drug permeating through the skin at different time intervals was calculated by equation (1):

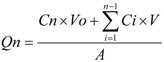
(1)
where Cn is the drug concentration of the receiver solution at each sampling time, Ci the drug concentration of the i (h) sample, and V_0_ and V the volumes of the receiver solution and the sample, respectively, A represents the skin surface area. The flux was calculated from the slope of the linear portion of the profile. All parameters were reported as the mean ± S.D. Statistical analysis was carried out by using analysis of variance (ANOVA). The level of significant was taken as P < 0.05. A correlation analysis was performed with the aid of the SPSS program, and correlation co-efficient were examined for significance (P < 0.05) by using Student’s t-test. 

## 4. Conclusions

In summary, the above experimental results show that different molecular weights of *N-*Arg-CS with different degrees of substitution have the potential to enhance the rate of adefovir transdermal delivery. In particular, the most effective transdermal enhancer is 2% (2 mg/mL) *N-*Arg-CS (MW 10 kDa) with 6% substitution degree. Chemically a complex of arginine and CS can significantly enhance the transdermal penetration compared to arginine and CS used singly, which was considered to be a result of strong interaction between positive and negative charge in *N-*Arg-CS and adefovir, then the guanidinium groups and amino groups of *N-*Arg-CS are strongly adsorbed on the cell membrane surfaces to open the tight junctions of the epithelia, allowing for the paracellular transport. These *N-*Arg-CS simulated arginine-rich cell penetration peptides have potential as a novel transdermal enhancer, although the mechanism of action needs further investigation.
